# Neurorehabilitation for cognitive and motor recovery: mechanisms, interventions, and translational advances

**DOI:** 10.3389/fneur.2026.1828447

**Published:** 2026-09-24

**Authors:** Yingchao Guan, Yanan Gao

**Affiliations:** Beijing Geriatric Hospital, Beijing, China

**Keywords:** frailty and post-stroke epilepsy, glial cell roles, innovative interventions for cognitive, mechanisms of neural plasticity, motor recovery

## Abstract

This narrative review synthesizes recent advances in neurorehabilitation, focusing on mechanisms of neural plasticity, glial cell function, and interventions for cognitive and motor recovery. We explore multidisciplinary approaches, including cognitive training, motor rehabilitation, neurostimulation, and regenerative therapies, highlighting their translational potential. Special attention is given to clinically relevant rehabilitation modifiers and complications, including spasticity, frailty, and post-stroke epilepsy, alongside strategies for enhancing quality of life and social reintegration. Emerging technologies such as brain-computer interfaces, virtual reality, and robotics are discussed for their role in personalized rehabilitation. This review emphasizes evidence-based, patient-centered interventions to enhance functional recovery and quality of life, identifying future directions for personalized neurorehabilitation across stroke survivors, elderly, and frail populations.

## 1 Introduction

Neurological disorders, including stroke, traumatic brain injury, and neurodegenerative diseases, affect approximately 3.4 billion people worldwide, constituting the leading cause of disability and the second leading cause of death globally (1, 2). It is estimated that one in three individuals could benefit from rehabilitation during illness or injury to enhance function or quality of life (3). For many incurable neurological conditions, comprehensive rehabilitation is vital for managing long-term disability. Approximately 60% of stroke survivors experience cognitive impairment within the first year post-onset, and a substantial proportion of stroke survivors separately experience persistent motor dysfunction (4). Neurorehabilitation improves physical function and quality of life, offering a cost-effective strategy to enhance functional outcomes and promote independence (1).

Modern neurorehabilitation adopts a multidisciplinary approach to address patients’ needs across multiple domains, including physical function, cognitive ability, emotional regulation, and social adaptation. Its application extends beyond stroke and traumatic brain injury to other neurological disorders, including multiple sclerosis and Parkinson disease, with rehabilitation strategies tailored to disease-specific functional impairments (5). Furthermore, population aging and the increasing prevalence of frailty are expanding the global demand for rehabilitation services. Accordingly, this review examines the biological basis of recovery and current strategies for cognitive and motor rehabilitation, with particular attention to neurostimulation, regenerative and technology-assisted approaches, clinically relevant complications, and future directions. This article was conducted as a narrative review. Relevant literature was identified through searches of PubMed/MEDLINE, Web of Science, and Scopus, with priority given to recent clinical guidelines, systematic reviews, meta-analyses, randomized trials, and representative mechanistic studies. Studies were selected according to their relevance to neural recovery mechanisms, clinical rehabilitation strategies, and translational advances; no formal risk-of-bias assessment or quantitative evidence synthesis was performed.

## 2 Biological basis of neurorehabilitation

### 2.1 Neural plasticity and functional recovery

Functional recovery following neurological injury is fundamentally dependent on experience-driven neural plasticity. This includes synaptic adaptation, axonal sprouting, dendritic remodeling, and neural circuit reorganization (6, 7). While these plastic changes support functional recovery, uncoordinated reorganization may lead to maladaptive outcome (6). Rehabilitation strategies grounded in the principles of neuroplasticity, particularly the “use it or lose it” theory—which posits that neural circuits not actively engaged in task performance may weaken—have become core to modern neurorehabilitation (8). Beyond behavioral practice alone, several external and physiological drivers can be applied to specifically promote cortical reorganization: non-invasive stimulation techniques such as repetitive transcranial magnetic stimulation (rTMS) and transcranial direct current stimulation (tDCS) modulate cortical excitability directly (see Section 4.1); neuromuscular electrical stimulation (NMES) paired with volitional movement reinforces sensorimotor pathways through synchronous afferent–efferent activity; and aerobic exercise upregulates brain-derived neurotrophic factor (BDNF), creating a molecular milieu that is more permissive to synaptic strengthening (see Section 3.2). These interventions operationalize the “use it or lose it” principle by providing a controllable, dosable stimulus for plasticity rather than relying on spontaneous experience-dependent change alone. For example, repetitive task training has been shown to enhance synaptic connectivity and improve motor function in stroke survivors (9).

### 2.2 Role of glial cells in recovery

Astrocytes undergo reactive astrogliosis following central nervous system injury, exerting dual roles by forming glial scars that inhibit axonal regeneration while also buffering glutamate and supporting neurogenesis (10). On one hand, their proliferation contributes to glial scar formation and inhibits axonal regeneration, thereby exacerbating tissue damage. On the other hand, astrocytes help buffer excess glutamate, reduce cerebral edema, and maintain homeostasis, exerting neuroprotective effects (11). In subacute to chronic stages, reactive astrocytes also facilitate angiogenesis, neurogenesis, and synaptic remodeling. These findings suggest that modulating astrocyte activity may represent a promising therapeutic target—by promoting a neuroprotective phenotype or enhancing glutamate uptake capacity (12). Moreover, metabolic dysfunction in astrocytes, such as impairments in the lactate shuttle mechanism, has been implicated in neurodegenerative diseases, potentially contributing to excitotoxic neuronal injury (13). Thus, targeting the metabolic and activation states of astrocytes may offer new strategies to delay neurodegeneration and enhance tissue repair.

Microglia, the principal immune cells of the central nervous system, exhibit highly dynamic and context-dependent responses after neurological injury. In acute brain injury, microglial activation may contribute to tissue damage through inflammatory signaling and oxidative stress, while also supporting debris clearance, trophic signaling, angiogenesis, and subsequent tissue remodeling (14, 15). These functions vary with disease context, anatomical region, and stage of recovery. Accordingly, the traditional binary classification into pro-inflammatory “M1” and anti-inflammatory “M2” phenotypes does not fully capture the heterogeneous microglial states observed *in vivo*. Therapeutic strategies should therefore aim to limit maladaptive inflammation while preserving repair-supportive functions rather than simply promoting a unidirectional phenotypic switch (16).

Oligodendrocytes are central to remyelination and neural repair. Following injury, oligodendrocyte precursor cells proliferate and differentiate into mature oligodendrocytes. Preclinical studies demonstrate that physical training and enriched environments significantly enhance oligodendrocyte precursor cell differentiation and adaptive myelination, leading to functional improvements (17). In demyelinating disorders such as multiple sclerosis, combining rehabilitation with strategies targeting oligodendrocyte precursor cells—particularly by modulating their interactions with astrocytes and microglia—has emerged as a promising research direction. Recent research has also highlighted the critical role of astrocyte-neuron coupling in the lateral habenula in mediating stress-induced depressive-like behaviors, suggesting potential glial targets for psychiatric neurorehabilitation (18). A schematic overview of the dual roles of astrocytes, microglia, and oligodendrocyte lineage cells in neural repair is shown in Figure 1.

**Figure 1:**
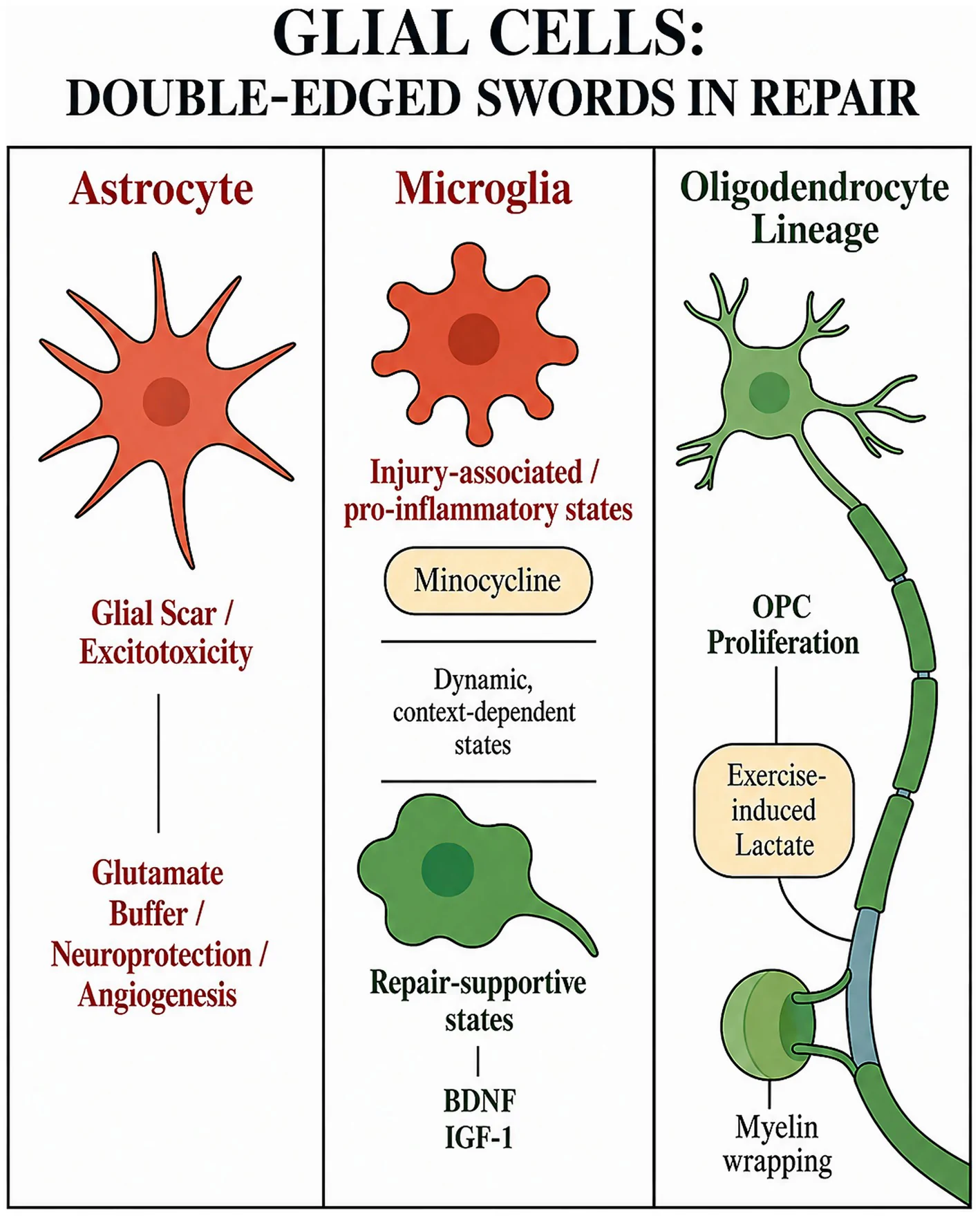
Glial cells: double-edged swords in neural repair. OPC, oligodendrocyte precursor cell; BDNF, brain-derived neurotrophic factor; IGF-1, insulin-like growth factor-1.

### 2.3 Therapeutic targeting of glial cells

Glial cells are increasingly recognized not as passive support elements, but as active regulators of neural repair. Based on this paradigm shift, several glia-targeted therapeutic strategies are under development: (1) astrocyte transplantation or administration of astrocyte-derived neurotrophic factors; (2) pharmacological modulation of microglia to regulate neuroinflammation; and (3) biomaterial-based engineering of the glial scar to support axonal regeneration (19, 20). A promising advance involves *in situ* reprogramming of reactive astrocytes into functional neurons, with encouraging preclinical results in animal stroke models (21). With continued insights into glial cell metabolism and signaling pathways, future interventions may achieve more precise modulation—enhancing the neuroprotective roles of glia in degenerative diseases, and optimizing their regenerative potential in neural repair (10), as summarized in Figure 2.

**Figure 2:**
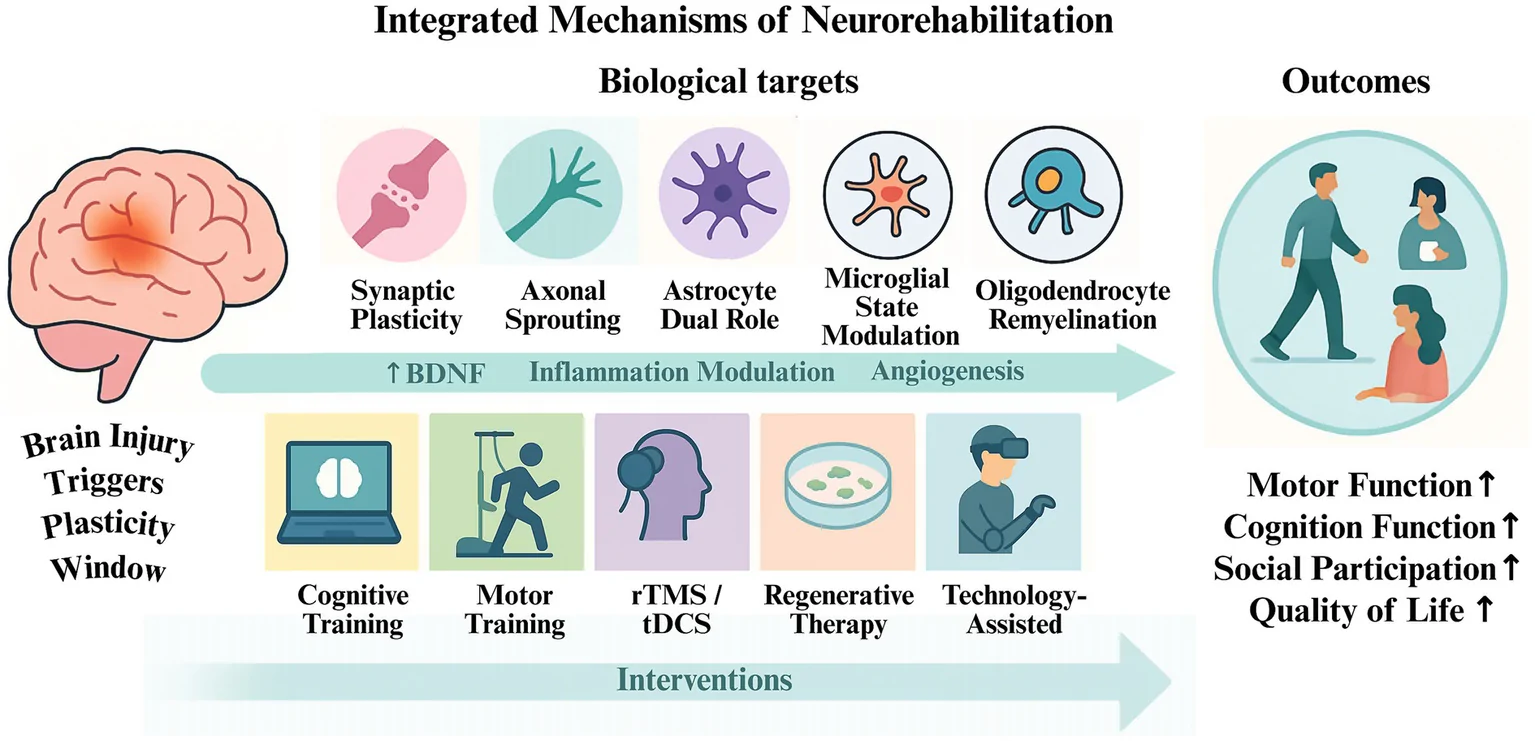
Integrated mechanisms of neurorehabilitation following brain injury. Neurorehabilitation integrates biological plasticity mechanisms and therapeutic interventions following brain injury. Key processes include synaptic plasticity, axonal sprouting, context-dependent glial modulation, and remyelination, together with BDNF signaling, inflammation modulation, and angiogenesis, contributing to functional and social recovery. BDNF, brain-derived neurotrophic factor; tDCS, transcranial direct current stimulation; rTMS, repetitive transcranial magnetic stimulation.

Importantly, glial responses vary across neurological diseases and stages of recovery; glial-targeted strategies should therefore be interpreted according to disease context and timing rather than as a uniform rehabilitation approach (10, 16).

## 3 Rehabilitation of cognitive and motor impairments

### 3.1 Cognitive rehabilitation

Cognitive rehabilitation facilitates the reorganization of brain function through systematic training programs grounded in principles of neural plasticity. Repetitive cognitive training has been shown to induce lasting neuroplastic changes, including increased brain volume, enhanced synaptic connectivity in targeted brain regions, and improved regional cerebral perfusion (22). In older adults, high-intensity cognitive interventions are significantly associated with microstructural brain remodeling and maintenance of cognitive performance (23).

Cognitive performance after neurological injury cannot be considered in isolation from co-occurring motor, sensory, and perceptual deficits, which frequently confound both the assessment and the rehabilitation of cognitive function (24, 25). Hemiparesis and impaired proprioception can limit a patient’s ability to complete motor-dependent cognitive assessments and computerized training tasks, while visual and somatosensory deficits (e.g., hemianopia, hemispatial neglect) can mimic, or compound, attentional and executive impairments (26). Perceptual deficits such as apraxia or agnosia further interfere with the execution of instrumental activities of daily living even when core cognitive processes are relatively preserved (27). Comprehensive cognitive rehabilitation programs therefore increasingly incorporate concurrent motor, sensory, and perceptual retraining, and clinicians are encouraged to interpret cognitive outcomes in the context of a patient’s sensorimotor status rather than treating cognition as an independent domain (28).

The efficacy of cognitive rehabilitation has been demonstrated across a range of neurological disorders. A recent randomized controlled trial reported that a four-week home-based computerized cognitive training program significantly improved attention, processing speed, and memory in stroke patients with cognitive impairment (18). Similarly, individuals with moderate-to-severe traumatic brain injury showed significant improvements across seven cognitive domains following structured cognitive rehabilitation (29). In patients with mild cognitive impairment or dementia, meta-analyses have confirmed that computerized cognitive training—particularly when supervised by professionals—yields substantial benefits in memory (especially verbal memory) and daily functioning (30, 31). However, the benefits of cognitive training do not always generalize: meta-analytic evidence indicates that gains in trained cognitive domains often show limited transfer to untrained cognitive domains and to daily-life functional activities, underscoring the need for training paradigms that explicitly incorporate functionally relevant, real-world tasks (32).

Emerging approaches now integrate cognitive training with other modalities, such as aerobic exercise or non-invasive brain stimulation (e.g., transcranial magnetic stimulation), to further augment neuroplasticity. Current evidence suggests that such multimodal interventions may enhance BDNF expression, thereby potentiating the effects of cognitive training (33, 34). Overall, cognitive rehabilitation represents a cornerstone of neurorehabilitation by leveraging the brain’s intrinsic capacity for plasticity and functional reorganization.

### 3.2 Motor training and functional restoration

Motor training promotes functional neurological recovery through multiple mechanisms: (1) upregulating BDNF expression to enhance synaptic plasticity and neurogenesis (35); and (2) stimulating angiogenesis to improve cerebral perfusion. Evidence indicates that even a single session of moderate-intensity aerobic exercise can directly and dose-dependently elevate serum BDNF levels in stroke survivors, with high-intensity interval training yielding even greater effects, demonstrating that rehabilitative exercise actively modulates this biomarker rather than merely correlating with its levels (36). These molecular changes correlate positively with motor learning capacity, and motor improvements are most pronounced when aerobic exercise is paired with task-oriented training (37).

Clinical data support the efficacy of structured exercise programs in restoring motor function. A meta-analysis of 12 studies involving 651 patients demonstrated that robot-assisted gait training combined with conventional rehabilitation significantly outperformed traditional therapy alone in improving lower limb motor function, balance, and walking endurance (38). Notably, high-intensity gait training (via treadmill or overground walking) was more effective than low-intensity training in enhancing walking speed. For upper limb rehabilitation, constraint-induced movement therapy and repetitive task practice have shown efficacy in restoring arm function (39). Overall, the current body of evidence supports a positive correlation between exercise intensity and functional gains, suggesting that high-intensity regimens should be prioritized when safely applicable (36).

The benefits of motor interventions in neurorehabilitation are multidimensional. Randomized controlled trials have shown that structured aerobic exercise can significantly improve attention, information processing speed, and executive function in individuals with chronic stroke (40). From a secondary prevention perspective, regular physical activity reduces the risk of recurrent stroke through mechanisms such as blood pressure regulation and improved glucose metabolism. Current clinical guidelines recommend at least 150 min of moderate-intensity aerobic activity per week for neurorehabilitation patients, with individualized modifications (e.g., seated aerobic training or hydrotherapy) to accommodate varying levels of physical function.

On a neurobiological level, exercise exerts its effects through two primary mechanisms: (1) modulation of neuroendocrine function and (2) enhancement of BDNF-mediated neuroplasticity. Clinically, exercise has been shown to alleviate post-stroke depressive symptoms and apathy (35). Importantly, improved cognitive function may, in turn, increase patient adherence to rehabilitation programs, creating a positive feedback loop.

Exercise-based rehabilitation models grounded in the empowerment paradigm include three core elements: (1) goal-directed interventions, such as setting personalized functional targets (e.g., “walking independently to the garden”) and providing motivational guidance (41); (2) self-management strategies, including education on the benefits of exercise and wearable device–based activity monitoring (42, 43); and (3) social support systems, such as community fitness groups and survivor-led adaptive sports programs. These comprehensive interventions not only enhance adherence but also support sustained functional improvement beyond formal therapy.

Motor interventions have been shown to significantly improve functional independence and quality of life. Systematic reviews have found that physical training effectively enhances walking endurance in stroke patients, while resistance training improves gait speed and balance without exacerbating spasticity (44). These physiological gains translate directly into improved activities of daily living, such as stair climbing, community mobility, and task endurance. Clinical studies also indicate that such functional improvements boost patients’ self-efficacy. Mechanistically, exercise yields integrative effects through the coordinated modulation of neuromuscular and cardiovascular systems. It is therefore an indispensable component of neurorehabilitation and synergizes effectively with other therapeutic modalities (45).

### 3.3 Spasticity management

Spasticity may interfere with voluntary movement, gait, positioning, pain control, hygiene, and participation in rehabilitation, but treatment should be driven by functional goals rather than muscle tone alone. Management should be individualized according to neurological etiology, severity, distribution, disease stage, and the presence of weakness or fixed contracture (46). Rehabilitation strategies include stretching, positioning, task-specific training, electrical stimulation, and vibration, with oral antispastic agents used mainly for more diffuse symptoms and botulinum toxin for clinically relevant focal or multifocal spasticity (46–48). Intrathecal baclofen or surgical interventions may be considered in selected patients with severe or refractory spasticity. Importantly, excessive tone reduction may not always be desirable, particularly when residual tone contributes to standing, transfers, or other functions (46).

## 4 Neurostimulation and regenerative therapies

### 4.1 Non-invasive brain stimulation

Repetitive transcranial magnetic stimulation (rTMS) is a non-invasive technique that modulates cortical excitability via magnetic pulses, capable of inducing long-term potentiation- or long-term depression-like plastic changes in the brain (7, 49). High-frequency rTMS (≥5 Hz) has been shown to enhance excitability in targeted cortical areas, whereas low-frequency rTMS (about 1 Hz) exerts inhibitory effects (7). rTMS has the potential to “trigger neural plasticity and enhance synaptic transmission,” a mechanism central to cortical reorganization after stroke. Clinically, rTMS has been widely used in stroke rehabilitation, with evidence supporting its efficacy in improving motor and cognitive functions, alleviating dysphagia (50), and treating post-stroke depression (51). Meta-analyses confirm that rTMS, when combined with conventional therapy, significantly improves upper limb motor function in stroke survivors (7). In addition, rTMS is an evidence-based treatment for major depression, exerting therapeutic effects by modulating emotion-related neural circuits (52).

Transcranial direct current stimulation (tDCS) modulates neuronal membrane potential through the application of low-intensity electrical current via scalp electrodes. Unlike methods that directly elicit action potentials, tDCS alters cortical excitability to create a permissive environment for neuroplasticity (49). In stroke rehabilitation, the anode is typically placed over the lesioned motor cortex to increase excitability, while the cathode is positioned contralaterally to reduce interhemispheric inhibition. However, findings across studies remain heterogeneous—some clinical trials report significant improvements in motor or language function when tDCS is combined with rehabilitation. A recent meta-analysis showed that tDCS significantly improved activities of daily living (as measured by the Barthel Index), especially in the acute phase, although effects on specific motor outcomes varied (53). Mechanistically, tDCS may exert its effects by modulating glutamate–GABA balance and facilitating synaptic plasticity related to learning (41). It is cost-effective, safe, and non-invasive, although optimizing stimulation parameters remains a key area of ongoing research.

Beyond conventional rTMS and tDCS, intermittent theta-burst stimulation (iTBS) and transcranial alternating current stimulation (tACS) are emerging approaches for rehabilitation-oriented neuromodulation. iTBS provides patterned magnetic stimulation capable of inducing relatively rapid changes in cortical excitability, whereas tACS aims to modulate endogenous neural oscillations. These techniques are increasingly being investigated as rehabilitation “primers” delivered before or alongside task-specific training, robotic therapy, or VR to facilitate activity-dependent plasticity. However, their incremental clinical benefit over rehabilitation alone remains uncertain because of heterogeneous protocols and relatively limited comparative evidence (54, 55).

### 4.2 Invasive brain stimulation

Deep brain stimulation is an invasive neuromodulation technique involving the implantation of electrodes in subcortical nuclei to deliver continuous electrical impulses. Deep brain stimulation has revolutionized the treatment of movement disorders. Clinical studies have shown that deep brain stimulation significantly alleviates core motor symptoms—tremor, rigidity, and bradykinesia—in patients with Parkinson’s disease, thereby improving mobility and quality of life (56). It is currently the standard of care for advanced Parkinson’s disease and essential tremor, with expanding indications for dystonia and obsessive-compulsive disorder (57). In neurorehabilitation, deep brain stimulation is being explored for the treatment of post-stroke pain and functional recovery in chronic stroke. Its therapeutic mechanism involves modulation of dysfunctional neural circuits—for example, stimulation of the motor thalamus or basal ganglia may reset abnormal oscillatory activity, facilitating motor control (56). With ongoing advances, the integration of adaptive (closed-loop) stimulation systems and advanced imaging-guided targeting is expected to further optimize outcomes (57). While deep brain stimulation remains an invasive technique, it provides valuable treatment options for refractory tremor and neuropsychiatric disorders. With advancements in precision medicine, future neuromodulation strategies are anticipated to become increasingly individualized, enabling real-time adaptation of stimulation parameters to optimize therapeutic outcomes.

### 4.3 Regenerative therapies

Regenerative medicine holds substantial promise in neurorehabilitation, particularly through stem cell transplantation for stroke and other central nervous system injuries. Current clinical trials predominantly utilize bone marrow-derived mononuclear cells, mesenchymal stem cells, or neural progenitor cells administered via intravenous or intra-arterial routes. These studies consistently affirm the safety of stem cell therapy, with minimal reports of serious adverse events (58). However, clinical efficacy remains inconclusive. While some small-scale trials have shown improvements in motor function and lesion volume reduction, meta-analyses indicate that these findings lack statistical significance and require validation through larger, double-blind randomized trials (59, 60). A meta-analysis on ischemic stroke confirmed the safety of stem cell therapy but could not establish its clinical efficacy (59).

Mechanistically, stem cells are believed to exert effects through paracrine secretion of growth factors and immunomodulatory signals, enhancing endogenous repair processes. Current research is focused on optimizing therapeutic parameters, including timing of administration (with subacute phases being potentially ideal), cell type selection, dosing, and delivery methods. The MULTISTEM (MASTERS) phase II trial showed that administration of allogeneic progenitor cells within 36 h post-stroke benefited specific patient subgroups, despite not meeting primary endpoints (61). The European RESSTORE trial also confirmed the safety of adipose-derived stem cells and suggested potential functional benefits in the subacute stage, although complete results are pending (62).

Beyond stem cell therapy, regenerative medicine encompasses a range of innovative approaches. Notably, the direct reprogramming of astrocytes into neurons via viral delivery of transcription factors (e.g., NeuroD1) has shown promise in rodent models, supporting functional recovery (21). This suggests a future in which endogenous glial cells may be harnessed for neuronal regeneration. Other emerging strategies include implantable biomaterial scaffolds loaded with stem cells or trophic factors, olfactory ensheathing cell transplantation, and neurotrophin infusion for spinal cord injury (63). Of particular interest, epidural spinal cord stimulation combined with intensive training has enabled some spinal cord injury patients to regain the ability to walk. Translating this model to stroke rehabilitation may enhance upper limb recovery (64–66).

The concept of “regenerative rehabilitation” emphasizes the integration of biological or bioelectronic interventions with physical rehabilitation to promote the functional integration of newly generated neural connections. Although most neuroregenerative technologies remain in experimental stages, their combination with established rehabilitation strategies may open new avenues for alleviating neurological impairments.

### 4.4 Technology-assisted rehabilitation

Technology-assisted rehabilitation represents a major innovation frontier in neurorehabilitation, integrating robotic devices, virtual reality (VR) systems, brain–computer interfaces (BCI), and tele-rehabilitation platforms. These technologies aim to enhance training intensity, personalize interventions, and extend access to care. A schematic overview of key technology-assisted rehabilitation modalities, including robotics, VR, brain–computer interfaces, and tele-rehabilitation, is presented in Figure 3.

**Figure 3:**
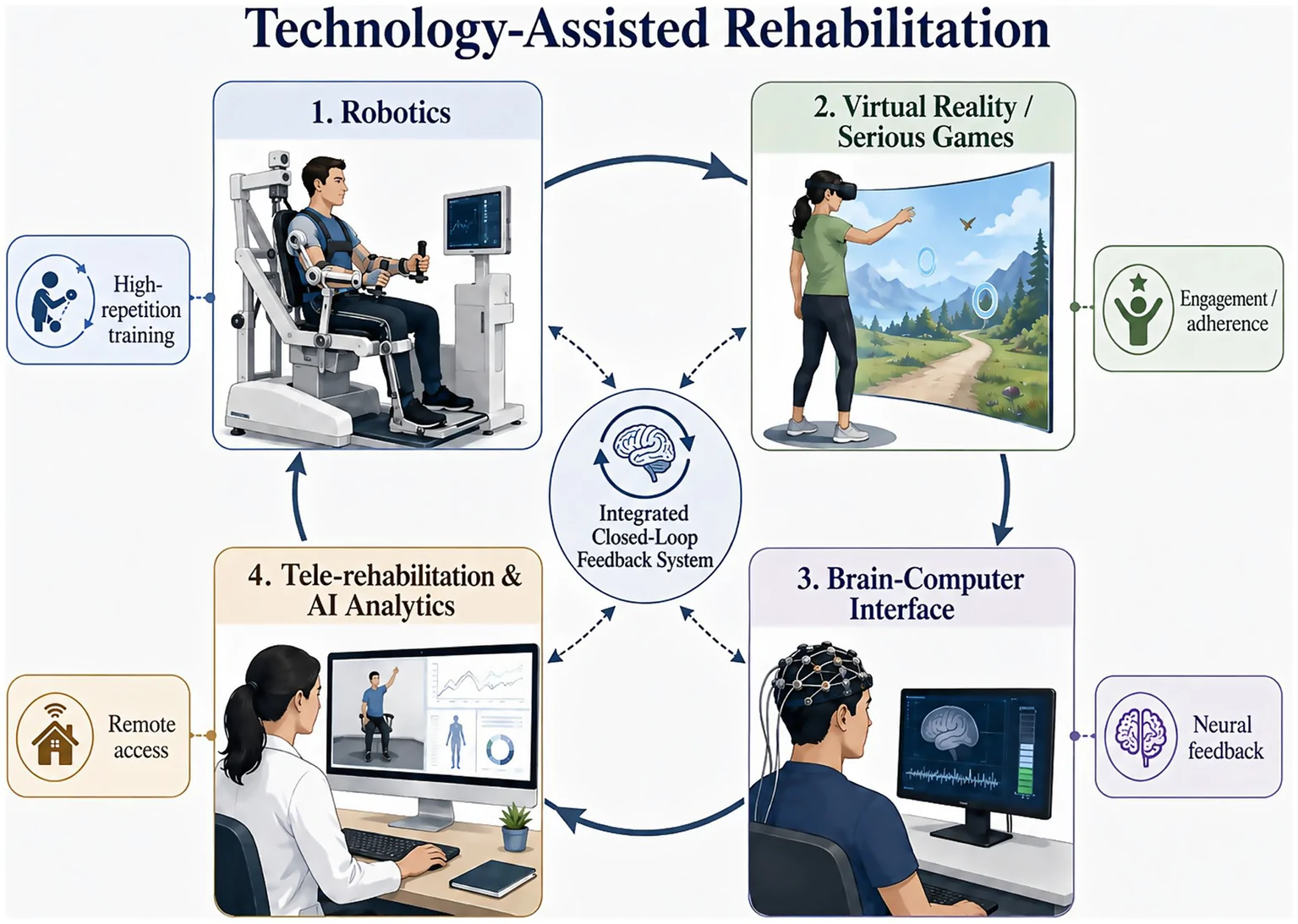
Technology-assisted rehabilitation landscape. Schematic overview of robotics, virtual reality/serious games, brain–computer interfaces, and tele-rehabilitation with AI analytics, integrated through a closed-loop feedback system.

#### 4.4.1 Robotic rehabilitation systems

Robotic rehabilitation systems can augment conventional neurorehabilitation by enabling high-intensity, repetitive, and precisely controlled training paradigms (67). These systems are typically divided into two categories: upper limb robots (exoskeletons or end-effectors targeting the shoulder, elbow, or wrist) and lower limb robots (gait trainers and wearable exoskeletons) (68). Clinical evidence for robotic therapy is encouraging but should be interpreted with appropriate caution. A meta-analysis of 34 trials reported statistically significant improvements in arm function and daily living abilities with robotic-assisted upper limb therapy compared with conventional therapy (69); however, the authors of that review themselves urged caution in interpreting these findings, and a more recent umbrella review concluded that, while robotics can produce measurable gains, effect sizes are frequently small—particularly for gait-related outcomes—and that statistically significant improvements do not necessarily translate into clinically meaningful functional change (69, 70). Likewise, robot-assisted gait training has been shown to enhance walking independence and balance in stroke patients, particularly when combined with conventional physical therapy (38). The therapeutic rationale nevertheless differs across neurological conditions. After stroke, robotics primarily supports intensive task-specific motor relearning; in spinal cord injury, it may additionally facilitate assisted standing, stepping, and mobility; and in Parkinson’s disease or multiple sclerosis, the emphasis is more often on gait, balance, endurance, and repetitive practice. Evidence in traumatic brain injury, cerebellar disorders, and peripheral neurological conditions, including Guillain–Barré syndrome, remains more limited. Robotic rehabilitation should therefore be interpreted according to disease, impairment, therapeutic goal, and stage of recovery rather than as a homogeneous intervention (71).

The core advantage of robotic systems lies in their ability to fine-tune assistive force or resistance, offer task-specific training (e.g., goal-oriented grasping, gait normalization), and deliver real-time biofeedback. In settings with limited therapist resources, upper-limb robotic devices can, in some configurations, extend training time with reduced direct supervision; this benefit does not, however, generalize uniformly across device types, and many robotic systems still require therapist supervision, particularly during gait training (70). Furthermore, by integrating gamified scenarios and VR, robotic systems maintain patient engagement. Recently developed wearable exoskeletons have enabled even severely paralyzed individuals to engage in standing and walking activities, with their application expanding from clinical to home environments. Technological trends point toward intelligent robotic systems with real-time adaptive control and soft robotics for enhanced ergonomic fit. Importantly, while robots can significantly increase training intensity, clinical decision-making and psychosocial support from therapists remain indispensable. The ideal rehabilitation model should leverage human–machine synergy (67).

#### 4.4.2 Virtual reality

VR can enhance neurorehabilitation outcomes through interactive, simulated environments, although the magnitude of benefit relative to conventional therapy varies by outcome and VR modality, and not every VR approach outperforms traditional rehabilitation. Systems range from immersive head-mounted displays to motion-tracked screen-based games (e.g., Wii/Kinect). The most authoritative synthesis to date, the 2025 Cochrane review by Laver and colleagues, found moderate- to low-certainty evidence that virtual reality and interactive video gaming are slightly more beneficial than alternative therapy approaches in improving upper limb function, balance, and activity limitation after stroke (72). Earlier meta-analyses similarly reported that VR outperforms conventional therapy in improving upper limb function, gait, and balance in stroke survivors (73, 74), but these more optimistic estimates should be read alongside the more conservative, higher-certainty Cochrane conclusions. Key advantages of VR include: (1) high-repetition training through gamified tasks (e.g., virtual fruit-catching), (2) safe simulation of daily life activities (e.g., crossing the street, grocery shopping), and (3) multisensory feedback that boosts training motivation. Studies also show cognitive gains (e.g., memory, executive function) when VR is combined with traditional therapy (75). Beyond conventional gamified tasks, VR can also incorporate creative activities such as virtual painting, music, or other interactive tasks that may support engagement, motivation, cognition, and emotional well-being; these applications should, however, be distinguished from formal art therapy (76). VR is also increasingly used in vestibular rehabilitation, where controlled visual environments can facilitate visual–vestibular integration, sensory reweighting, gaze stabilization, postural control, and graded exposure to visually complex environments. Evidence remains heterogeneous, and superiority over well-delivered conventional rehabilitation has not been consistently established (77). In balance training, VR environments effectively guide weight-shifting and obstacle avoidance. In one network meta-analysis, combined VR and robotic therapy ranked favorably for selected balance and gait outcomes, although heterogeneity and indirect comparisons limit conclusions regarding comparative superiority (78). During the COVID-19 pandemic, VR and game-based platforms played a critical role in telerehabilitation, increasing compliance for home-based training. As costs decline and usability improves, these interactive tools may become more widely integrated into neurorehabilitation, although their comparative effectiveness and cost-effectiveness require further evaluation.

#### 4.4.3 Brain-computer interfaces

BCI technology represents a cutting-edge approach that decodes neural signals (e.g., EEG) in real time and translates them into control commands for external devices (79). Clinically, BCI systems can detect a patient’s motor intent (e.g., intention to open the hand) and activate functional electrical stimulation or robotic devices accordingly. This closed-loop paradigm not only enables functional compensation for paralyzed limbs but may also promote neuroplastic changes in the motor cortex. Evidence supports the efficacy of combining BCI with robotics or functional stimulation in improving motor outcomes after stroke (68, 80). Although currently limited to research settings, BCI holds promise as a neuroplasticity-enhancing modality in clinical rehabilitation.

#### 4.4.4 Tele-rehabilitation and AI

Tele-rehabilitation technologies have expanded access to care, offering clinical outcomes comparable to in-person visits (81), and are particularly valuable for patients living in remote or rural areas where access to specialized rehabilitation services is otherwise limited. These systems integrate wearable sensors and smart devices for real-time activity monitoring, enabling personalized and data-driven treatment planning. Three-dimensional printing facilitates rapid, precise customization of orthotic devices. Artificial intelligence and machine learning are being investigated across four major areas of neurorehabilitation: assessment and monitoring, prediction of recovery and prognosis, treatment personalization, and clinical decision support. Algorithms may integrate clinical, sensor, imaging, and electrophysiological data to quantify patient performance, estimate recovery trajectories, and adapt rehabilitation intensity or device assistance. However, much of the current evidence remains retrospective or proof-of-concept, with important limitations including external validation, algorithmic bias, interpretability, privacy, and clinical workflow integration. AI should therefore currently be regarded primarily as a tool to augment, rather than replace, rehabilitation assessment and clinical decision-making (82–84).

The convergence of stem cell therapy, regenerative medicine, robotic systems, VR, and BCI technologies is driving neurorehabilitation toward a more efficient, personalized, and participatory future. These emerging approaches are primarily designed to complement traditional therapy by augmenting clinical capacity and improving patient adherence. As clinical evidence accumulates, some of these technologies may be incorporated into standard care. Future stroke rehabilitation may involve multimodal strategies—initiating with biological interventions (e.g., stem cells or trophic factors), followed by AI-guided robotic therapy and home-based VR training. Achieving this vision will require further research to optimize technology integration, evaluate cost-effectiveness, and ensure accessibility in resource-limited settings. The rapid evolution of rehabilitation technologies offers new hope for improving outcomes in patients with neurological impairments. To facilitate comparison across the major approaches discussed above, Table 1 summarizes their current clinical role, level of clinical maturity, and principal limitations.

**Table 1:** Clinical maturity and current role of major neurorehabilitation approaches.

Approach	Population/Current role	Evidence maturity	Main limitation
Task-specific motor/cognitive rehabilitation	Broad neurological populations; core rehabilitation	Established–moderate	Variable transfer to daily function
rTMS/tDCS	Mainly stroke; adjunctive neuromodulation	Moderate	Protocol and response heterogeneity
iTBS/tACS	Mainly stroke; rehabilitation priming	Emerging	Limited comparative evidence
Robotic rehabilitation	Stroke/SCI; emerging in PD/MS; increased training dose/repetition	Moderate, disease-dependent	Modest effects, cost/device dependence
Virtual reality	Stroke and vestibular disorders; interactive motor/balance/cognitive training	Low–moderate	Heterogeneous platforms and outcomes
BCI/regenerative therapies	Mainly stroke/SCI; restoration or repair strategies	Early translational	Limited robust clinical evidence
AI/ML	Cross-condition; assessment, prognosis, personalization, decision support	Emerging	Validation, bias and implementation

Evidence maturity is presented qualitatively to indicate the current stage of clinical translation and does not represent a formal GRADE assessment.

## 5 Special populations and complication management

### 5.1 Frailty rehabilitation strategies

Frailty is a clinical syndrome characterized by a decline in physiological reserve and an impaired ability to cope with external stressors (85). It is notably prevalent among elderly individuals following a stroke. Studies suggest that 23%–25% of stroke patients meet frailty diagnostic criteria, with 40%–45% classified as pre-frail, though variability across studies underscores the need for standardized assessments (85, 86). This high prevalence underscores the importance of incorporating frailty assessment into post-stroke care to inform prognosis and guide individualized rehabilitation strategies. Epidemiological data indicate that frail stroke patients are often older women and are at higher risk of poor outcomes: they exhibit greater levels of disability, are more likely to require institutional care (46.9% vs. 18.5%), receive fewer active treatments, and experience longer hospital stays (87). During rehabilitation, frailty substantially limits the effectiveness of standard therapies, manifesting as delayed cognitive recovery, increased risk of delirium, suboptimal response to conventional interventions, and minimal gains in daily functioning (86).

Clinical practice suggests that individualized rehabilitation programs are essential for frail patients. Key strategies include extended rehabilitation duration, enhanced nutritional support, and integration of comprehensive geriatric assessment to prevent further functional decline. Management protocols emphasize: (1) comprehensive geriatric assessment-based evaluations; (2) optimized nutritional intake—especially protein, caloric, and vitamin D supplementation; (3) structured physical activity programs, particularly progressive resistance training combined with balance exercises; and (4) rationalization of polypharmacy (87).

Evidence indicates that even modest functional improvements—such as a 10% increase in muscle strength—can significantly enhance independence (88). Supportive interventions include early mobilization, fall prevention training, home environment modifications, and the use of assistive devices. Although frail individuals may achieve smaller absolute improvements, the relative benefit is substantial (effect size d = 0.42) (87). Multidisciplinary frailty management has been shown to reduce complications and promote successful community reintegration.

Recent research in frailty management focuses on prehabilitation programs designed to improve physiological reserves in high-risk individuals, the development of pharmacological adjuncts such as anabolic agents and anti-inflammatory drugs to enhance muscle function and reduce systemic inflammation, and precision rehabilitation models that apply tiered interventions based on the severity of frailty. These strategies reflect a growing shift toward proactive, individualized care to optimize outcomes in vulnerable populations (89, 90).

### 5.2 Management of post-stroke epilepsy

Post-stroke epilepsy (PSE) is a common neurological complication, with incidence varying by stroke subtype. Clinical data indicate that PSE occurs in approximately 5%–10% of patients with ischemic stroke and 10%–20% with hemorrhagic stroke (91). Large-scale cohort studies have confirmed PSE incidence rates of 6.4% in ischemic stroke and 12.4% in hemorrhagic stroke (92). The risk of seizure is highest within the first year post-stroke but may persist long-term. Major risk factors include cortical involvement (as opposed to subcortical lesions), hemorrhagic stroke, higher stroke severity, and younger age (93). The underlying pathophysiology involves glial proliferation, iron deposition, and maladaptive neural network remodeling. Notably, early seizures occurring within 1–2 weeks post-stroke are predictive of chronic epilepsy in approximately 30%–50% of cases (94).

PSE can negatively affect the rehabilitation process and overall quality of life in multiple ways. Seizures themselves can cause temporary functional regression and increase the risk of falls, while also leading to activity restrictions such as driving prohibitions. Additionally, adverse effects of antiepileptic drugs—including fatigue, attentional deficits, and cognitive impairments—may further hinder recovery (95, 96). Studies have shown that patients with PSE have higher mortality rates compared to those without seizures, suggesting that epilepsy not only reflects the severity of the underlying brain injury but may directly interfere with rehabilitation outcomes. Psychologically, seizures often induce fear and anxiety in patients and caregivers, reducing adherence to rehabilitation protocols. Therefore, once seizure control is achieved, structured patient education and psychological support are essential to rebuild confidence in treatment (97). Proper management and integrated intervention for PSE are crucial to minimizing its negative impact on rehabilitation and long-term outcomes.

Current clinical guidelines do not recommend primary prophylaxis of epilepsy in stroke patients (i.e., using antiepileptic drugs in patients with no history of seizures) (98). Evidence shows that prophylactic antiepileptic drugs neither significantly reduce seizure incidence nor improve clinical outcomes. Moreover, unnecessary antiepileptic drug use may increase the risk of adverse effects, particularly in complex stroke cases. The primary focus in PSE management is on controlling vascular risk factors—such as blood pressure—and closely monitoring for early seizure activity. For patients experiencing early seizures, newer-generation antiepileptic drugs such as levetiracetam are preferred due to their favorable tolerability, minimal drug interactions, and limited cognitive side effects (99).

Research is increasingly focused on neuroprotective strategies, including experimental agents targeting post-stroke neuroinflammation and excitotoxic glutamatergic signaling. However, no pharmacological intervention has yet been validated for routine clinical prevention (100). For patients with confirmed PSE, comprehensive management should integrate standardized antiepileptic drug therapy with active rehabilitation. While most patients respond well to monotherapy, some may require combination regimens. Rehabilitation teams should also implement seizure precaution measures, such as avoiding photosensitive stimuli and adjusting high-risk training activities. Additionally, patients should be counseled on modifiable lifestyle factors that may lower seizure thresholds (e.g., sleep deprivation). Psychosocial support is equally important and includes counseling services and participation in support groups to improve quality of life (91). Importantly, seizures do not preclude successful rehabilitation. With proper management, many patients with PSE can achieve meaningful functional recovery. Ongoing research is investigating the potential of early interventions—such as anti-inflammatory and immunomodulatory therapies—to prevent the development of epilepsy after stroke, which may open new avenues for improving long-term outcomes.

## 6 Multidimensional outcome evaluation and social reintegration

### 6.1 Patient-centered goals and QoL assessment

Modern neurorehabilitation increasingly emphasizes not only functional recovery but also social participation and quality of life (QoL) as core outcome indicators. Evidence indicates that successful rehabilitation enhances not only muscle strength and cognitive performance but also patients’ ability to resume meaningful roles in family, community, and valued activities (101). Patient-centered goal setting—such as resuming employment, participating in family life, or engaging in hobbies—has been shown to significantly enhance treatment engagement and adherence. Studies confirm that collaborative goal-setting strategies empower patients, promote autonomy, and are positively associated with improved functional outcomes (102).

Research further indicates that structured rehabilitation interventions significantly improve QoL in patients with stroke and other neurological conditions. Inpatient rehabilitation enhances QoL by increasing functional independence (103), and longitudinal studies have shown progressive QoL improvements following 3 to 12 months of sustained rehabilitation (104). These improvements stem from multiple factors, including motor recovery, increased ability to perform activities of daily living, and enhanced social participation. Notably, even when residual disabilities persist, rehabilitation can positively impact mental health by fostering a sense of autonomy and hope (105).

### 6.2 Community integration and functional Independence

Community reintegration is a fundamental goal of neurorehabilitation, with key strategies including: (1) mobility training (e.g., walking on uneven terrain, wheelchair skills); (2) communication training for individuals with aphasia or cognitive impairment, aimed at improving functional dialogue and transactional interactions; and (3) instrumental activities of daily living training such as cooking, shopping, and money management (106–108).

Occupational therapists play a crucial role in enhancing employment outcomes and social engagement (109). Vocational rehabilitation programs support individuals with brain injuries in re-entering the workforce or participating in volunteer activities. Driving rehabilitation services—through adaptive training or vehicle modification—further promote independence (110). Standardized tools, such as the Community Integration Questionnaire, have validated the effectiveness of outpatient and community-based programs in enhancing reintegration (111). Multidisciplinary teams support community re-entry by addressing depressive symptoms, fatigue, and other psychosocial barriers. Interventions such as psychological counseling and caregiver education contribute to a holistic rehabilitation model that aligns with the principle of not merely prolonging life, but improving its quality. This person-centered approach reinforces the overarching goal of neurorehabilitation: restoring individuals to meaningful, autonomous participation in society (112).

## 7 Future perspectives

The interventions summarized above span substantially different levels of clinical maturity, from established rehabilitation practices to emerging and experimental approaches. The field is increasingly exploring how selected therapies can be combined, sequenced, and individually tailored rather than applied in isolation. Emerging evidence supports layering neurostimulation onto behaviorally driven training to capitalize on activity-dependent (Hebbian) plasticity—the principle that neurons or circuits activated together become more strongly connected (“fire together, wire together”)—so that rTMS, tDCS, or neuromuscular electrical stimulation delivered concurrently with, or immediately before, task-specific motor or cognitive training may prime cortical excitability and amplify training-induced reorganization more than either approach alone (7, 41). Trials combining aerobic exercise with cognitive training or non-invasive stimulation report synergistic increases in BDNF expression and greater functional gains than single-modality interventions (36, 37). Looking forward, rehabilitation decision-making is expected to move from a uniform, therapy-by-therapy approach toward biomarker- and lesion-informed selection: matching patients to specific interventions—and deciding when to switch to, or add, a therapy—based on lesion location and severity, circulating biomarkers such as BDNF and inflammatory markers, and neurophysiological readouts (e.g., motor-evoked potential presence, EEG connectivity), with the explicit goal of maximizing functional status for each individual rather than applying a one-size-fits-all protocol.

Neurorehabilitation has evolved into a comprehensive, evidence-based discipline that significantly improves clinical outcomes in patients with neurological impairments. The field integrates a wide spectrum of interventions, ranging from conventional physical therapies to cutting-edge biomedical technologies. At its core is the principle of facilitating neuroplasticity through targeted stimulation, demonstrating that functional recovery is possible even in the chronic phase after injury (6). Interdisciplinary collaboration—encompassing neurology, rehabilitation medicine, psychology, and engineering—forms the foundation for delivering holistic care, echoing the person-centered philosophy of evaluating patients’ cognitive, behavioral, emotional, and physical functions as a whole (113).

Technological innovation is driving the advancement of personalized rehabilitation. Artificial intelligence is being applied to clinical data to optimize treatment planning (83); home-based VR systems and tele-rehabilitation platforms are expanding access to services (114); and wearable devices enable real-time activity monitoring (115). Neuroscience research has also revealed novel strategies for enhancing memory consolidation through stimulation during specific sleep phases (116). Pharmacological approaches such as the use of selective serotonin reuptake inhibitors to modulate neuroplasticity are currently under clinical investigation (117). In the field of regenerative medicine, neural stem cell transplantation and gene therapy are being investigated in combination with conventional rehabilitation, although their incremental clinical benefit remains to be established.

Despite these advancements, significant challenges remain, including the high cost of technologies like robotics and BCI, limited scalability in low-resource settings, and variable patient access due to digital literacy or socioeconomic barriers. Addressing these issues requires cost-effective solutions, scalable tele-rehabilitation models, and equitable policy frameworks to ensure widespread adoption. Ensuring equitable access to rehabilitation services remains a global challenge. The World Health Organization’s “Rehabilitation 2030” initiative emphasizes the need to integrate rehabilitation into health systems, strengthen the rehabilitation workforce, and promote community-based interventions, particularly in resource-limited settings. Telehealth has emerged as a promising strategy to bridge geographical and infrastructural barriers, enhancing healthcare accessibility for underserved populations. However, disparities persist due to factors such as digital literacy and access to technology (116, 117). Neurorehabilitation is undergoing a transformative shift. Three trends define this evolution: precision-based interventions informed by multi-omics data, AI integration, and personalized service models (82, 84). Ultimately, rehabilitation should aim not simply to help patients survive their injury, but to help them rebuild a life they find worth living—a goal that will require continued investment in both scientific discovery and compassionate, individualized care.

## 8 Conclusion

Neurorehabilitation has become an integrated discipline combining neuroscience, clinical rehabilitation, and engineering technologies, demonstrating a significant impact on functional recovery, quality of life, and social reintegration for individuals with neurological disorders. This review highlights the evolution of neurorehabilitation into a multidisciplinary field that strategically leverages neural plasticity, glial cell modulation, and technological innovation to enhance outcomes. Key advances include the integration of cognitive and motor training, neurostimulation, regenerative therapies, and emerging tools such as VR and robotics. With the advent of artificial intelligence, telemedicine, and precision rehabilitation, the field is advancing rapidly toward more efficient, intelligent, and patient-centered approaches. Future efforts should prioritize the enhancement of translational research, optimization of resource allocation, and promotion of the widespread standardization and accessibility of rehabilitation technologies to deliver continuous, comprehensive care for neurological populations.
